# Molecular identification and genetic characteristics of *Cryptosporidium* spp., *Giardia duodenalis*, and *Enterocytozoon bieneusi* in human immunodeficiency virus/acquired immunodeficiency syndrome patients in Shanghai, China

**DOI:** 10.1186/s13071-023-05666-8

**Published:** 2023-02-04

**Authors:** Yanyan Jiang, Li Liu, Zhongying Yuan, Aiqin Liu, Jianping Cao, Yujuan Shen

**Affiliations:** 1 National Institute of Parasitic Diseases, Chinese Center for Disease Control and Prevention (Chinese Center for Tropical Diseases Research), NHC Key Laboratory of Parasite and Vector Biology, WHO Collaborating Centre for Tropical Diseases, National Center for International Research on Tropical Diseases, 200025 Shanghai, China; 2 Shanghai Public Health Clinical Center, Shanghai Medical College, Fudan University, 201508 Shanghai, China; 3 Department of Parasitology, Harbin Medical University, Harbin, 150081 China

**Keywords:** *Cryptosporidium* species, *Giardia duodenalis*, *Enterocytozoon bieneusi*, Human immunodeficiency virus, Genotype, Subtype, Zoonotic transmission

## Abstract

**Background:**

Opportunistic infections are a ubiquitous complication in human immunodeficiency virus (HIV)/acquired immunodeficiency syndrome (AIDS) patients. *Cryptosporidium* spp., *Giardia duodenalis*, and *Enterocytozoon bieneusi* are common opportunistic intestinal pathogens in humans. In China, despite the number of HIV/AIDS patients being extremely large, only a few studies have investigated opportunistic infections caused by intestinal pathogens in this patient population. The aims of this study were to elucidate the occurrence and genetic characteristics of *Cryptosporidium* spp., *G. duodenalis*, and *E. bieneusi* in HIV/AIDS patients.

**Methods:**

We collected fecal specimens from 155 HIV/AIDS patients (one from each patient). All of the specimens were examined for the presence of the pathogens by genotyping using polymerase chain reaction and sequencing of the small subunit ribosomal RNA gene for *Cryptosporidium* spp.; the triosephosphate isomerase, β-giardin and glutamate dehydrogenase genes for *G. duodenalis*; and the internal transcribed spacer region of the rRNA gene for *E. bieneusi*. The *Cryptosporidium*-positive specimens were further subtyped by polymerase chain reacion and sequencing of the 60-kDa glycoprotein gene.

**Results:**

Six (3.9%), three (1.9%), and eight (5.2%) HIV/AIDS patients were positive for *Cryptosporidium* spp., *G. duodenalis*, and *E. bieneusi*, respectively. No statistical differences were observed in occurrence rate between the groups by gender, clinical symptom (diarrhea), and CD4^+^ cell count. Four *Cryptosporidium* species were identified: *Cryptosporidium hominis* (*n* = 2), *Cryptosporidium parvum* (*n* = 1), *Cryptosporidium meleagridis* (*n* = 1), and *Cryptosporidium andersoni* (*n* = 2). Furthermore, two *C. hominis* subtypes (IeA12G3T3 and IaA28R4) were detected. Three *G. duodenalis*-positive specimens were successfully amplified and sequenced at the triosephosphate isomerase and β-giardin loci, which led to the identification of assemblages C and B, respectively. Seven genotypes (D, Type IV, EbpC, Peru11, EbpD, A, and I) were identified in *E. bieneusi*-positive specimens.

**Conclusions:**

Our findings should increase awareness of AIDS-related opportunistic intestinal pathogens, and indicate the need for routine examination in clinical practice for the detection of *Cryptosporidium* spp., *G. duodenalis*, and *E. bieneusi*. Homology analyses of the three intestinal pathogens at the nucleotide and/or amino acid levels indicated their zoonotic potential.

**Graphical Abstract:**

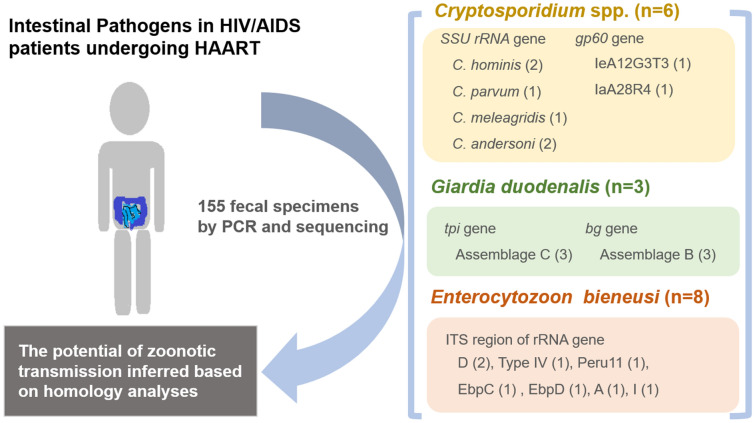

## Background

Acquired immunodeficiency syndrome (AIDS) is an important public health concern worldwide, and deprives hundreds of thousands of people of their lives each year. AIDS is the fourth major cause of human death and the leading cause of death due to infectious disease [1]. Serious opportunistic infections (OIs) are the prominent cause of death in patients with advanced AIDS, with many such infections posing a serious threat to the health of human immunodeficiency virus (HIV)/AIDS patients [2]. *Cryptosporidium* spp., *Giardia duodenalis* (syn. *Giardia intestinalis* or *Giardia lamblia*), and *Enterocytozoon bieneusi* are common opportunistic intestinal pathogens in humans [3]; their global prevalence in HIV/AIDS patients is 14.0%, 5.0% and 9.2%, respectively [4, 5]. Infections caused by these pathogens are mainly characterized by diarrhea, which can become chronic or life-threatening in immunocompromised individuals, particularly in HIV/AIDS patients [5–7]. The number of patients living with HIV has continued to increase across the globe, from approximately 2 million people in 2016 to approximately 38 million people in 2019 [8, 9]. Early diagnosis of infectious diseases is pivotal to decreasing the occurrence of their severe clinical consequences in this patient population.

*Cryptosporidium* spp., *G. duodenalis* and *E. bieneusi* are found in animals as well as in humans. In humans, they are transmitted via the fecal–oral route, either directly (human-to-human/animal contact) or indirectly (via ingestion of water or food contaminated by human or animal feces) [10, 11]. The role of water and food in the epidemiology of infections caused by these pathogens is now well recognized. Many outbreaks of these infections have been reported worldwide, with waterborne outbreaks being more common (respectively > 524 and > 26 for cryptosporidiosis [12], > 344 and > 38 for giardiasis [12], and one and two for microsporidiosis caused by *E. bieneusi* [13–15]). Based on their clinical and public health importance, *Cryptosporidium* spp., *G. duodenalis*, and *E. bieneusi* are included in the Environmental Protection Agency Contaminant Candidate List of microbes of concern for waterborne transmission (https://www.epa.gov/ground-water-and-drinking-water/national-primary-drinking-water-regulations). In addition, in 2014, *Cryptosporidium* spp. and *G. duodenalis* were ranked, respectively, as the fifth and the 11th most important foodborne parasites worldwide by a joint Food and Agriculture Organization and World Health Organization of the United Nations study [16].

The application of molecular genotyping and subtyping tools has considerably enhanced our ability to trace sources of contamination or infection and to elucidate transmission routes of pathogens. To date, at least 44 *Cryptosporidium* spp. and 120 genotypes have been identified. *Cryptosporidium hominis* and *C. parvum* are highly prevalent (> 90%) in human cases of cryptosporidiosis worldwide [17]. Further, eight assemblages (A–H) of *G. duodenalis* have been characterized, and assemblages A and B are responsible for the vast majority (99%) of human cases of giardiasis [18]. *Enterocytozoon bieneusi* is a genetically complex species. To date, at least 685 *E. bieneusi* genotypes, belonging to 13 distinct genetic groups, have been identified [19]. At least 106 *E. bieneusi* genotypes are found in humans [20], and genotypes D, EbpC, and Type IV are commonly reported in patients with *E. bieneusi* infection [21].

In China, the number of people infected with HIV is high and shows an upward trend. At the end of 2020, there were 1.1 million people living with HIV in China [22]. Epidemiological studies on *Cryptosporidium* spp., *G. duodenalis*, and *E. bieneusi* in this population have been conducted across several provinces and municipalities of China, with reported prevalences of *Cryptosporidium* spp., *G. duodenalis* and *E. bieneusi* of 0.7–60.0% [23], 1.3–16.2% [24, 25], and 5.7–11.6% [26], respectively. However, no associated data are available on infections caused by these opportunistic intestinal pathogens in HIV/AIDS patients in Shanghai (one of the four direct-controlled municipalities). *Cryptosporidium* spp., *G. duodenalis*, and *E. bieneusi* are routinely detected in local patients (adults and children) with diarrhea [27–31]. Studies have also reported the occurrence of these pathogens in animals (e.g., livestock, companion animals, and zoo animals) [32], and the environment (e.g., in source and tap water, combined sewerage systems and wastewater treatment plant effluent) [33–38].

Thus, we investigated the rates of occurrence of the intestinal pathogens *Cryptosporidium* spp., *G. duodenalis*, and *E. bieneusi* in HIV/AIDS patients and determined their genetic characteristics at the genotype and/or subtype levels by using molecular techniques. The sources of contamination or infection by *Cryptosporidium* spp., *G. duodenalis*, and *E. bieneusi* were traced, and the transmission routes of these pathogens assessed by homology analysis of their nucleotide or amino acid sequences.

## Methods

### Participants

In total, 155 HIV/AIDS patients (of whom only four had diarrhea) were enrolled in the study from July 2013 to March 2017. All of the patients were followed at Shanghai Public Health Clinical Center, and had been on highly active antiretroviral therapy (HAART). All of the patients were adults, aged from 18 to 64 years; 141 (91.0%) were males and 14 (9.0%) were females. At the time of sampling of the fecal specimens, the CD4^+^ cell count was > 200 cells/μL in 92 of the patients and < 200 cells/μL in 63 of the patients.

### Specimen collection and DNA extraction

Fecal specimens were collected from all of the patients (one per patient), immediately transferred to the laboratory in a cooler containing ice packs, and subsequently stored at − 80 °C for molecular detection of *Cryptosporidium* spp., *G. duodenalis*, and *E. bieneusi*. Genomic DNA was directly extracted from an approximately 180- to 200-mg fecal specimen using the QIAamp DNA Stool Mini Kit (QIAgen, Hilden, Germany), in accordance with the manufacturer’s instructions. DNA was eluted in 200 μL AE buffer and stored at − 20 °C until needed.

### Polymerase chain reaction amplification

The DNA preparations were screened for the presence of *Cryptosporidium* spp., *G. duodenalis*, and *E. bieneusi* via nested polymerase chain reacion (PCR) and sequence analysis. *Cryptosporidium* spp. were genotyped and subtyped by amplifying the small subunit ribosomal RNA [SSU rRNA; approximately 830 base pairs (bp)] [39] and 60-kDa glycoprotein (*gp60*) genes (approximately 800–850 bp) [40], respectively. *Giardia duodenalis* was genotyped by amplifying the triosephosphate isomerase (*tpi*) gene (approximately 530 bp) [41], the β-giardin (*bg*) gene (approximately 510 bp) [42], and the glutamate dehydrogenase gene (approximately 530 bp) [43]. *Enterocytozoon bieneusi* was identified and genotyped by PCR analysis of an approximately 410-bp region of the rRNA gene covering the entire internal transcribed spacer (ITS) region (243 bp) [44]. PCR was performed with positive controls (chicken-derived *Cryptosporidium baileyi* DNA for *Cryptosporidium* spp., cattle-derived assemblage E DNA for *G. duodenalis*, and deer-derived genotype BEB6 DNA for *E. bieneusi*) as well as negative controls (DNase-free water). All the secondary PCR products were subjected to electrophoresis in a 1.5% agarose gel, and were visualized by staining the gel with ethidium bromide.

### Sequencing and data analyses

All of the PCR amplicons of expected size were purified and then directly sequenced using the corresponding primers on an ABI PRISMTM 3730 DNA Analyzer (Applied Biosystems, Carlsbad, CA) with a BigDye Terminator v3.1 Cycle Sequencing kit (Applied Biosystems). Sequencing data accuracy was validated by sequencing in both directions. Nucleotide sequences were subjected to Basic Local Alignment Search Tool (BLAST) searches (http://www.ncbi.nlm.nih.gov/blast/) and then aligned and analyzed with reference sequences downloaded from GenBank, using Clustal X 1.83 (http://www.clustal.org/) to determine the genotypes and subtypes of *Cryptosporidium-*, *G. duodenalis-*, and *E. bieneusi-*positive specimens. If the obtained nucleotide sequences were different from published sequences, two separate PCR amplicons of the same DNA preparation were sequenced to ensure accuracy.

### Statistical analysis

Statistical analysis was performed with SPSS 26.0. Pearson chi-square and Fisher’s exact tests were used to determine statistical significance.

## Results

### Occurrence of *Cryptosporidium spp.*, *G. duodenalis* and *E. bieneusi*

PCR and sequence analyses revealed that, of the 155 HIV/AIDS patients, six, three, and eight patients were positive for *Cryptosporidium* spp., *G. duodenalis*, and *E. bieneusi*, respectively. *Enterocytozoon bieneusi* (5.2%) was more prevalent than *Cryptosporidium* spp. (3.9%) and *G. duodenalis* (1.9%). One patient had a mixed infection with *G. duodenalis* and *E. bieneusi*.

Females had a higher rate of infection with *Cryptosporidium* than males (7.1% vs. 3.6%). *Giardia duodenalis* and *E. bieneusi* were only found in males. All *Cryptosporidium*- and *E. bieneusi*-positive cases were of non-diarrheal patients. *G. duodenalis* was identified in both diarrheal and non-diarrheal patients. However, no statistical difference in occurrence rate of diarrhea was found between them (Table 1). *Cryptosporidium* spp., *G. duodenalis*, and *E. bieneusi* were detected in patients with a CD4^+^ cell count of < 200 cells/μL and in patients with a CD4^+^ cell count of > 200 cells/μL, and no significant association was found between CD4^+^ cell count and infections caused by these pathogens.

**Table 1:** Occurrence of *Cryptosporidium* spp., *Giardia duodenalis* and *Enterocytozoon bieneusi* in human immunodeficiency virus (HIV)/acquired immunodeficiency syndrome (AIDS) patients

Groups	Examined (*n*)	*Cryptosporidium* spp.		*G. duodenalis*		*E. bieneusi*	
		Positive [*n* (%)]	χ2/*P*-value	Positive [*n* (%)]	χ2/*P*-value	Positive [*n* (%)]	χ2/*P*-value
Gender							
Male	141	5 (3.6)	0.44/0.51	3 (2.1)	0.57/0.45	8 (5.7)	1.56/0.21
Female	14	1 (7.1)		0 (0)		0 (0)	
Diarrhea							
Yes	4	0 (0)	0.32/0.57	1 (25.0)	2.41/0.12	0 (0)	0.43/0.51
No	151	6 (4.0)		2 (1.3)		8 (5.3)	
CD4^+^ T-lymphocyte count (cells/μL)							
< 200	63	4 (6.4)	1.75/0.19	2 (3.2)	0.86/0.35	3 (4.8)	0.03/0.85
> 200	92	2 (2.2)		1 (1.1)		5 (5.4)	
Total	155	6 (3.9)		3 (1.9)		8 (5.2)	

### *Cryptosporidium* genotyping and subtyping

Sequence analysis of the SSU rRNA gene revealed the presence of four *Cryptosporidium* spp.: *Cryptosporidium hominis* (*n* = 2), *Cryptosporidium parvum* (*n* = 1), *Cryptosporidium meleagridis* (*n* = 1), and *Cryptosporidium andersoni* (*n* = 2). None of the SSU rRNA gene sequences had been previously described (GenBank accession numbers MT757967–MT757972). Table 2 shows the results of the homology analyses. DNA specimens characterized as originating from *C. hominis*, *C. parvum*, or *C. meleagridis* were further subjected to subtyping by sequence analysis of the *gp60* gene. However, only two of these specimens, from *C. hominis*, were successfully subtyped and identified, namely IaA28R4 (GenBank accession no. OM212052) and IeA12G3T3 (GenBank accession no. OM212051).

**Table 2:** Homology analysis of the small subunit ribosomal RNA gene sequences of *Cryptosporidium*-positive specimens

Species (*n*)	Accession nos.^a^ (*n*)	Accession nos.^b^ (host)/homology
*Cryptosporidium hominis* (2)	MT757967 (1)	ON023862 (rhesus monkey)/99.50%
	MT757968 (1)	MK990042 (human)/97.92%
*Cryptosporidium parvum* (1)	MT757969 (1)	AY204230 (human)/99.88%
*Cryptosporidium meleagridis* (1)	MT757970 (1)	KT151551 (chicken)/99.88%
*Cryptosporidium andersoni* (2)	MT757971 (1)	KF826309 (human)/94.64%
	MT757972 (1)	MK982465 (calf)/99.75%

^a^Accession nos. indicating the sequences obtained in the present study

^b^Accession nos. indicating reference sequences deposited in the GenBank database that have the highest similarity with those obtained in the present study

### *Giardia duodenalis* genotyping

Three *G. duodenalis-*positive specimens were successfully amplified and sequenced at the *tpi* and *bg* loci, and all of them showed mixed infections of assemblages C (at the *tpi* locus) and B (at the *bg* locus). Three of the *tpi* gene sequences of assemblage C had not been previously described. However, at the amino acid level, the *tpi* sequences showed 100% similarity with the sequences of dog-derived *G. duodenalis*: OM212053 and OM212054 were identical to KY979493, and OM212055 was identical to MW561663. In contrast, three *bg* gene sequences of assemblage B were identical and shared 100% similarity with the sequence of a squirrel monkey-derived *G. duodenalis* (GenBank accession no. KJ888974). Table 3 summarizes the results of the homology analyses.

**Table 3:** Homology analysis of the *tpi* and *bg* genes of *Giardia duodenalis*-positive specimens at the nucleotide and amino acid levels

Target gene	Assemblage (*n*)	GenBank accession nos.^a^	GenBank accession nos.^b^ (host)	Homology	Codon/amino acid
*Tpi*	C (1)	OM212053	KY979493 (dog); HG970114 (dog)	100%	
	C (1)	OM212054	KY979493 (dog); HG970114 (dog)	99.39%	G(G → T)G/G → V
	C (1)	OM212055	MW561663 (dog)	98.80%	G(G → T)G/G → V; G(G → T)G/G → V
*Bg*	B (3)	OM212056	KJ888974 (squirrel monkey)	100%	

^a^GenBank accession nos. indicating the nucleotide sequences obtained in this study

^b^GenBank accession nos. indicating the published nucleotide sequences with high similarity to the nucleotide sequences obtained in this study

### *Enterocytozoon bieneusi* genotyping

Based on sequence analysis of the ITS region, seven genotypes of *E. bieneusi* were identified: D (*n* = 2), EbpC, TypeIV, Peru11, A, and EbpD (one each) of group 1, and I (*n* = 1) of group 2.

## Discussion

HIV/AIDS patients are at high risk of OIs [45], which are a ubiquitous complication in patients with advanced AIDS. In China, despite the increasing number of patients with HIV/AIDS, little information is available on the occurrence of OIs in these patients, and in particular those OI caused by intestinal pathogens. We report here, to our best knowledge for the first time, the presence of *Cryptosporidium* spp., *G. duodenalis*, and *E. bieneusi* in HIV/AIDS patients in Shanghai, China.

The occurrence rates of *Cryptosporidium* spp., *G. duodenalis*, and *E. bieneusi* were 3.9%, 1.9%, and 5.2% in the HIV/AIDS patients, respectively, which are lower than those reported by most previous studies, particularly those from African countries. For example, the rates of *Cryptosporidium* spp., *G. duodenalis* and *E. bieneusi* in HIV-infected patients were reported to be as high as 79.0% in Nigeria [46], 40.7% in Uganda [47], and 76.9% in Uganda [48], respectively. These differences could be related to the progressive introduction of HAART and the National Free Antiretroviral Therapy Program initiated in 2002 by the Chinese government. In fact, since the introduction of HAART, a marked reduction has been seen in the occurrence of *Cryptosporidium* spp., *G. duodenalis*, and *E. bieneusi* in HIV/AIDS patients. The occurrence ratios between AIDS patients without and with HAART were 15.5 (3.1%/0.2%) for cryptosporidiosis in Australia and 10 European countries [49], 1.2 (6.7%/5.5%) for giardiasis in India, Ethiopia, and Cameroon [5], and 1.5 (13.8%/9.2%) for microsporidiosis caused by *E. bieneusi* in Guangxi, China [50]. 

Another reason for the lower occurrence rates of *Cryptosporidium* spp., *G. duodenalis*, and *E. bieneusi* in this study could be that almost all of the patients were non-diarrheal. Diarrhea is the most common clinical symptom associated with intestinal pathogen infections, particularly in immunocompromised individuals. Previous studies have confirmed a significant relationship between infections caused by *Cryptosporidium* spp., *G. duodenalis*, and *E. bieneusi* and the occurrence of diarrhea in HIV/AIDS patients. In India, AIDS patients with diarrhea reportedly showed a higher prevalence of these pathogens than those without diarrhea (*Cryptosporidium* spp., 46.0% vs. 8.0%; *G. duodenalis*, 16.0% vs. 8.0%; and microsporidiosis caused by *E. bieneusi* and *Encephalitozoon intestinalis*, 10% vs. 0%) [51]. Similar results have been reported for HIV/AIDS patients receiving antiretroviral therapy. In an investigation of diarrhea-associated etiologic agents in 164 HIV/AIDS patients in Kenya, *Cryptosporidium* spp., *G. duodenalis*, and *E. bieneusi*, detected by light microscopy, were found to be more prevalent in patients with diarrhea than in those without diarrhea (*Cryptosporidium* spp*.*, 16.0% vs. 6.0%; *G. duodenalis*, 10.0% vs. 3.0%; microsporidia, 10.0% vs. 2.0%) [52]. These findings should raise awareness in clinical practice of OIs caused by intestinal pathogens in HIV/AIDS patients, specifically in those with diarrhea. Early clinical intervention should reduce the occurrence of serious clinical consequences related to OIs.

In the present study, sequence analysis of the SSU rRNA gene led to the identification of *C. hominis*, *C. parvum, C. meleagridis*, and *C. andersoni*. *Cryptosporidium hominis* and *C*. *parvum* have the highest prevalences of the genus* Cryptosporidium* globally, and are responsible for > 90% of all human cases of *Cryptosporidium* infection/cryptosporidiosis [17]. *Cryptosporidium meleagridis* is the third most prevalent species of the genus infecting humans [17]. In China, *C. hominis* (127/265, 47.9%) and *C. parvum* (44/265, 16.6%) are the most prevalent species of the genus, and are responsible for 64.5% of all human cases of *Cryptosporidium* infection [23, 53], followed by *C*. *andersoni* (59/265, 22.3%) [12]. In a molecular epidemiological investigation of *Cryptosporidium* in diarrheal patients from southern Assam, India, *C. andersoni* was reported to be the predominant species, accounting for 79.6% of 98 *Cryptosporidium*-positive cases [54]. To date, *C. andersoni* has been found to be responsible for 148 human cases (including those identified in this study) of *Cryptosporidium* infection/cryptosporidiosis, with the vast majority (96.6%) of cases being reported in developing countries, including China, Myanmar, India, Iran, and Malawi [12]. It is notable that *C. andersoni* is a common species in ruminants, particularly in adult cattle [17]. However, the extent of its zoonotic transmission remains to be determined because of the absence of multilocus sequence typing (MLST) data for human-derived *C. andersoni*. MLST subtypes and the population genetic structure of animal-derived *C. andersoni*, including those from cattle, sheep, horses, golden takins, monkeys, camels, ostriches, and hamsters, have been analyzed in China [23]. Future studies need to employ MLST analysis of human-derived *C. andersoni* to facilitate elucidation of the sources of infection/contamination with *C. andersoni* and increase our understanding of its transmission dynamics.

Sequence analysis of the *gp60* gene has been widely used to subtype and track the transmission of zoonotic *Cryptosporidium* spp. and their genotypes [17]. Only two subtypes (IaA28R4 and IeA12G3T3) of *C. hominis* were identified in the present study, of which subtype IaA28R4 is an emerging *C. hominis* subtype in humans in China. To date, this subtype has only been identified in human cases of cryptosporidiosis in the USA (62 cases) [55] and Sweden (two cases) [56]. Subtype IeA12G3T3 has been previously reported in HIV/AIDS patients in China [57], and it was also detected in raw wastewater/sewage in wastewater plants in four cities in China, including Shanghai [36, 38]. Moreover, subtype IeA12G3T3 has been found in humans in Vietnam [58], Jamaica [59], Qatar [60], and Slovakia [61]. The data on *C. hominis* subtypes that are currently available indicate that IaA28R4 and IeA12G3T3 are rarely detected in humans and are restricted to certain countries.

Sequence analysis of the *bg* gene led to identification of assemblage B in three *G. duodenalis*-positive specimens. Among the six assemblages (A–F) of *G. duodenalis* found in humans, A and B are the most common worldwide, and B is responsible for more infections than A. In China, only three assemblages (A–C) have been identified in humans. In Myanmar, 70% (221/287) and 17.4% (50/287) of human cases of *G. duodenalis* infections were attributable to assemblages A and B, respectively [12]. Meanwhile, sequence analysis of the *tpi* gene identified assemblage C in three identical *G. duodenalis-*positive specimens, indicative of mixed infection with assemblages B and C. The increasing use of multilocus genotyping tools in epidemiological studies of *G. duodenalis* has led to more cases of mixed infections involving different assemblages being found in humans and animals [e.g., assemblages A and B (*n* = 15) in 54 *G. duodenalis*-positive asymptomatic immigrants in Qatar [62] and assemblages A and E (*n* = 4), B and E (*n* = 1), and C and D (*n* = 1) in 214 *G. duodenalis-*positive animal specimens in northeastern China] [63].

The homology analysis indicated that three *bg* gene sequences of assemblage B were identical. The same *bg* gene sequence has been previously described in squirrel monkeys from China [64]. Three different *tpi* gene sequences of assemblage C obtained in the present study have not been previously described. However, at the amino acid level, the *tpi* sequences showed 100% similarity with those derived from dogs: OM212053 and OM212054 were identical to KY979493 (China), and OM212055 was identical to MW561663 (Thailand). The results of the homology analysis indicated the zoonotic potential of assemblages B and C detected in the present study in patients from Shanghai. Although we identified assemblage C in the present study, it is usually found in canines, and only occasionally in humans. To date, assemblage C has been found in humans in Brazil [65], Egypt [66], Slovakia [67], and China [27]. In China, assemblage C has been found to be responsible for human cases of giardiasis only in Shanghai [27].

Sequence analysis of the ITS region revealed seven genotypes (D, Type IV, EbpC, Peru11, EbpD, A, and I) in the *E. bieneusi*-positive specimens. Among these, genotypes D, EbpC, and Type IV of group 1 have been most frequently identified in humans and numerous animal species worldwide, and display a low level of host specificity and zoonotic or cross-species transmission potential [11]. In China, the genotypes D, EbpC, and Type IV have been most commonly found in humans (ranked first, second, and fifth, respectively, according to number of cases of *E. bieneusi* infection) [68], and in at least 22, 12 and seven animal species, respectively [69]. Genotypes Peru11 and EbpD are also frequently detected in humans, and both of these show zoonotic potential; however, their host ranges (five and two animal species for genotypes Peru11 and EbpD, respectively) are smaller than those of genotypes D, EbpC, and Type IV (28, 16, and 9, respectively) [11]. In China, seven cases and one case, respectively, of microsporidiosis have been attributed to genotypes Peru11 and EbpD [68]. Genotype Peru11 has also been found in five non-human primate species and genotype EbpD in black-capped capuchins and pigs [69]. Genotype A is the most prevalent in humans globally [11]. This genotype was previously only found in humans, and was indicative of person-to-person transmission of *E. bieneusi* [26], but has since been detected in captive baboons in Kenya [70] and in dogs in Spain [71]. In China, besides its detection in HIV/AIDS patients in the present study, this genotype has also been detected in children [72]; however,  to date, no studies have reported genotype A in animals. Genotype I of group 2 was initially considered to be cattle-specific due its frequent detection in cattle [73]. *E. bieneusi* has been found in animal species such as pigs, deer, takins, cats, meerkats, rabbits, and bats, as well as in diarrheal children in China [69, 74]. These findings indicate that genotype I may be of public health concern due to its zoonotic potential. 

This study has some limitations. The number of analyzed specimens was small, and the number of positive specimens was low. In accordance with the One World—One Health Manhattan Principles, we believe that future molecular studies on the epidemiology of *Cryptosporidium* spp., *G. duodenalis*, and *E. bieneusi* need to be conducted using a sufficient number of human specimens, a greater variety of animal hosts and environmental specimens, to assess and validate the zoonotic potential of these pathogens. The types of data thus collected should facilitate the development of efficient control strategies for the management and prevention of OIs in HIV/AIDS patients.

## Conclusions

Herein we report the occurrence and genetic characteristics of *Cryptosporidium* spp., *G. duodenalis*, and *E. bieneusi* in HIV/AIDS patients in Shanghai, China. Our findings should increase awareness of opportunistic intestinal pathogens in AIDS patients, and indicate the importance in clinical practice of routine examination of patients for the detection of the common pathogens discussed here. The high diversity of *Cryptosporidium* spp. and the rare *gp60* subtypes of *C. hominis* identified in this study possibly reflect the genetic characteristics of *Cryptosporidium* endemic to China. Mixed infections with different assemblages were indicated by identification of assemblages B and C in three identical *G. duodenalis-*positive specimens. Seven zoonotic genotypes of *E. bieneusi*, including genotypes D, EbpC, Type IV (high frequency), Peru 11, EbpD, and A (commonly found), and I (rarely found), were also identified. Homology analysis of nucleotide and/or amino acid sequences of these pathogens revealed their zoonotic potential.

## Data Availability

The representative nucleotide sequences obtained in the present study were deposited in GenBank database under the following accession nos.: MT757967–MT757972 (*Cryptosporidium*), OM212053–OM212056 (*G. duodenalis*).

## Abbreviations

AIDS: Acquired immunodeficiency syndrome
*bg*: β-giardin
BLAST: Basic Local Alignment Search Tool
HAART: Highly active antiretroviral therapy
HIV: Human immunodeficiency virus
ITS: Internal transcribed spacer
OI: Opportunistic infection
SSU rRNA: Small subunit ribosomal RNA
*tpi*: Triosephosphate isomerase
